# Positive impacts of perceived social support on prosocial behavior: the chain mediating role of moral identity and moral sensitivity

**DOI:** 10.3389/fpsyg.2023.1234977

**Published:** 2023-10-16

**Authors:** Qiangqiang Li, Gengdan Hu

**Affiliations:** ^1^School of Educational Science, Jiangsu Second Normal University, Nanjing, China; ^2^Department of Psychology, School of Humanities, Tongji University, Shanghai, China; ^3^Clinical Research Center for Mental Disorders, Shanghai Pudong New Area Mental Health Center, School of Medicine, Tongji University, Shanghai, China

**Keywords:** perceived social support, prosocial behavior, moral identity, moral sensitivity, college student

## Abstract

The relationship between mental health and perceived social support has been well-established in previous studies. While previous research indicates that perceived social support is related to prosocial behavior, the mechanisms underlying this relationship remain unclear. In order to address this gap, a recent study investigated the mediating effects of moral identity and moral sensitivity on the relationship between perceived social support and prosocial behavior. Specifically, the study surveyed 978 college students using a questionnaire to examine the relationship between these variables. The results of the study showed that, after controlling for gender and age, perceived social support, moral identity, and moral sensitivity were significantly and positively correlated, all of which were also significantly and positively related to prosocial behavior. In addition, the study found that perceived social support was able to influence prosocial behavior through both independent mediation by moral identity and moral sensitivity, as well as through chain mediation. The findings of this study contribute to our understanding of the factors that shape prosocial behavior and offer suggestions for promoting such behavior in individuals.

## 1. Introduction

Individuals frequently engage in prosocial activity, which entails deeds that help other individuals. Examples include helping others through donations of money, organs, and time (Eisenberg et al., 1995). Such actions are assumed to result from people’s moral views, feelings, and ideals. Prosocial activity is crucial for fostering one’s own mental health because it fosters ties with individuals and society as a whole (Simpson and Willer, 2015). Furthermore, engaging in prosocial behavior is believed to impact individuals’ perception of their life’s purpose (Klein, 2017), enhance their feelings of happiness (Martela and Ryan, 2016), and shape their social interactions (Layous et al., 2012). Therefore, given the significance of prosocial activity in enhancing individual mental health, it is crucial that we investigate the factors that affect prosocial behavior as well as the psychological mechanisms that underlie this behavior.

### 1.1. Perceived social support and prosocial behavior

Prosocial behavior, which involves acts intended to benefit others, is often observed during interpersonal interactions. According to Social Exchange Theory, when individuals perceive more social support, they also provide more support to others (Cropanzano, 2005). Empirical studies have also shown that high levels of perceived social support significantly contribute to individuals’ prosocial behavior (Li et al., 2019). According to research conducted by Cirelli et al. (2014), interpersonal experiences can serve as a catalyst for the occurrence of prosocial behavior. However, when it comes to predicting prosocial behavior, individuals’ perceived subjective support (i.e., perceived social support) appears to be a more reliable indicator than objective support from the environment (You et al., 2022). Objective social support pertains to the actual respect, understanding, and support that an individual receives from others (Langford et al., 1997). In contrast, subjective social support refers to the level of satisfaction an individual experiences with the support available to them, as well as their perception of the extent to which they are supported externally (Langford et al., 1997; Zhang et al., 2023). The latter emphasizes the significance of self-perceptions in social support, which more accurately reflects an individual’s internal psychological state and is thus a more effective predictor of prosocial behavior.

In the realm of mental health and social adjustment, perceived social support has a direct impact (Yildirim et al., 2023), while prosocial behavior is a manifestation of a healthy psychological state and a high level of social adjustment. For example, previous studies have shown perceived social support significantly predicted resilience, satisfaction with life (Yildirim and Green, 2023) and emotional well-being (Yildirim et al., 2023), all of which are strongly associated with prosocial behavior. Thus, a heightened sense of social support can directly contribute to an increase in prosocial behavior. Referring to the Buffering Hypothesis (Cohen and Wills, 1985), it suggests that a high level of perceived social support can aid college students in forming positive perceptions, effectively coping with stressors in various aspects of life (Yildirim and Green, 2023), reducing the impact of negative life events similarly, social support as a psychological resource can be very effective in helping refugees to gain a sense of belonging and in contributing to a higher level of life satisfaction, and forming adaptive social networks, which lays the foundation for the development of prosocial behavior. On the other hand, social support pertains to psychological assistance or material support such as care, respect, and needs from family parental supports can be effective in mitigating mental health problems among adolescents living in disadvantaged areas (Alshehri et al., 2020), friends (Mahon and Yarcheski, 2016), group organizations, and other members. It is a crucial personal resource that plays an integral role in maintaining and promoting physical and mental well-being (Yildirim and Aziz, 2023). According to previous research, individuals who value positive interpersonal relationships and close connections within their organization tend to experience a strong sense of inclusion, leading to prosocial behavior (Twenge et al., 2007). Furthermore, a supportive social environment promotes the development and practice of prosocial behavior (de Guzman et al., 2013). Although earlier studies have shown a correlation between perceived social support and prosocial behavior, as demonstrated by Pfeiffer et al. (2016), there is a need for empirical evidence to investigate the underlying mechanisms. Hence, researchers must examine the relationship between perceived social support and prosocial behavior. In light of this, this study puts forward the hypothesis that perceived social support is a positive predictor of prosocial behavior (H1).

### 1.2. Moral identity as a mediating variable

Moral identity is a fundamental component of an individual’s self-concept, which is developed and structured based on a specific set of moral attributes. This framework, known as moral cognitive schema, measures an individual’s belief in the significance of moral qualities like honesty and kindness to their sense of self (Aquino and Reed, 2002). In essence, moral identity is a reflection of an individual’s moral values and beliefs and is deeply ingrained in their psychological makeup. Therefore, it can be said that moral identity is a crucial concept that plays a vital role in shaping an individual’s behavior and decision-making processes, particularly in situations that require moral judgment. According to the self-model theory (Blasi, 1984), an individual has the natural tendency to behave in ways consistent with his or her self-concept. Therefore, when individual identity is centered on morality, one will engage in prosocial behavior consistent with one’s moral self. Empirical studies support this notion. For example, Aquino et al. (2009) found that an individual has the desire to act in a consistent manner to their self-concept, which motivates an individual to engage in prosocial behavior. Prior research has indicated that individuals with elevated moral identity are more likely to engage in prosocial conduct (Kaur, 2020). Additionally, a meta-analysis conducted by Hertz and Krettenauer (2016) also found that moral identity was significantly and positively linked to predominantly prosocial behavior. Specifically, moral identity consists of two dimensions: moral internalization and moral symbolization (Aquino and Reed, 2002). Internalization of moral identity refers to the core level of an individual’s moral qualities in their self-concept, namely the importance of moral qualities to the self. Symbolization of moral identity refers to an individual’s tendency to demonstrate their moral qualities through behavior. On one hand, moral identity internalization was found to be a strong predictor of self-sacrifice, giving, and helping behavior. Furthermore, this dimension was deemed a relatively stable predictor of prosocial behavior. On the other hand, moral identity symbolization was more closely associated with overt, highly self-presented prosocial behavior. Individuals with high moral identity symbolization were found to exhibit more prosocial behavior in situations related to their moral image maintenance, self-presentation, and positive feedback from others. To conclude, individuals with heightened moral identity possess a strong inclination to align their self-concept and behavior with their moral identity. Failure to act accordingly in situations where prosocial behavior is appropriate might result in psychological distress (Winterich et al., 2013).

Previous studies on perceived social support have focused on perceived social support for psychological health, but little attention has been paid to the role of moral identity for perceived social support and prosocial behavior among college students. According to the social cognitive model of moral behavior proposed by Aquino et al. (2009), the extent to which moral identity is internalized can impact behavior. In fact, research has shown that perceived social support has a positive correlation with the acquisition of actual socially supportive behaviors (French et al., 2018). These behaviors are related to providing social support which is considered morally significant (Heerde and Hemphill, 2018). The acquisition of these morally relevant experiences contributes to the importance of moral schemas in the self (Aquino and Reed, 2002), thereby enhancing the individual’s internalization of moral identity, exhibiting more prosocial behavior. Additionally, according to the broaden-and-build theory of positive emotions, positive emotions promote the recovery of individuals’ psychological resources and lead to the construction of lasting personal resources. Research suggests that individuals who perceive high levels of social support tend to be more confident (Freeman and Rees, 2010). The acquisition of confidence makes individuals more likely to engage in self-promotion. This characteristic may lead individuals who perceive high levels of social support to be more inclined to exhibit their moral qualities and demonstrate higher levels of moral identity. In other words, perceiving high levels of social support can enhance individuals’ sense of moral identity, and because individuals with high moral identity are more likely to exhibit their moral qualities and seek the approval of others (Jennings et al., 2015), they are more likely to engage in prosocial behavior. Thus, moral identity could act as a mediator between perceived social support and prosocial behavior.

Consequently, from the social cognitive perspective, moral identity is a self-concept or self-schema composed of moral traits associated with moral behavior, which is stored in memory as a knowledge structure (Shao et al., 2008). Perceived social support can strengthen the internalization and representation of individuals’ moral identity, thus demonstrating more prosocial behavior. Therefore, based on the aforementioned, this study suggests hypothesis: Moral identity mediates the role between perceived social support and prosocial behavior (H2).

### 1.3. Moral sensitivity as a mediating variable

Moral sensitivity is a cognitive capacity that enables individuals to recognize moral aspects of a situation and respond appropriately to them. It involves the mental ability to interpret a situation from a moral perspective (Rest, 1982). Research indicates that heightened moral sensitivity contributes to the development of individuals’ ability to identify and manage moral and moral issues, and leads to increased moral conduct (Fowler et al., 2009). Conversely, a deficiency or absence of moral sensitivity can result in moral indifference and ignorance (Pedersen, 2009). Therefore, given the crucial role that moral sensitivity plays in personal growth and social interactions, investigating the connection between perceived social support, moral sensitivity, and prosocial behavior is of significant importance.

Some researchers have explored the impact of various factors on moral sensitivity from the perspectives of external context (Bredemeier and Shields, 1994) and internal personality traits. However, there is little research on the influence of social relationship networks on moral sensitivity, which is essentially formed in the social relationship network of interpersonal interaction. Based on this, this study aims to explore the impact of perceived social support on moral sensitivity from the perspective of social relationship networks, and the role and mechanism of moral sensitivity in the relationship between perceived social support and prosocial behavior, based on the social information processing theory.

Social information processing theory proposes that individuals use previously acquired semantic concepts, schemata, and past experiences to interpret new information. The procedure of establishing perceived social support, which refers to the caring, respectful, and aiding behavior that individuals receive from relatives, friends, or other groups (Wyer and Srull, 1986), comprises of a vast array of principles and frameworks concerning the support and refusal of social interactions (Mankowski and Wyer, 1997). Moral problems usually occur in interpersonal relationships (Jia and Krettenauer, 2017), and the identification of such predicaments necessitates individuals to amalgamate concepts and frameworks connected to social interactions. Consequently, in keeping with the social information processing theory, the concepts and frameworks supplied by perceived social support concerning care and support, rejection and harm in social interactions can furnish individuals with prior experience in assessing ethical issues, thereby augmenting their moral sensitivity. Moreover, Xiang et al. (2020) revealed that social support can have a positive impact on moral sensitivity. Based on this, this study suggests that perceived social support can positively predict an individual’s moral sensitivity (H3).

Moral sensitivity is positively related to prosocial behavior (Eisenberg, 2000). Moral sensitivity is one of the essential components guiding adaptive prosocial behavior patterns. Individuals more sensitive to moral problems, usually make prosocial decisions (Garrigan et al., 2018). Take nursing as an example, health care professionals with more moral sensitivity are more capable of appreciating situations that moral dilemma and making prosocial decisions directed at benefiting the patient. The importance of reinforcing such behavior in humanized care has also been emphasized (Suazo et al., 2020).

Prosociality is a multidimensional construct (Israel et al., 2015), which, in addition to the behavior mentioned above, has an affective dimension including social support, as an individual’s emotional experience and satisfaction with being respected, supported, and a tendency to understand and behave pro-socially. Perceiving social support can affect moral sensitivity. On one hand, according to Aquino et al.’s (2009) social cognitive model of moral behavior, the higher the degree of internalization of moral identity, the more important the moral schema occupies in the working self-concept, which enables people to quickly interpret their own and others’ actions from a moral perspective, that is, having higher moral sensitivity. Therefore, individuals who perceive high levels of social support show greater sensitivity to moral issues. On the other hand, research has shown that individuals who perceive high levels of social support are more confident (Freeman and Rees, 2010), and the acquisition of confidence makes them more likely to showcase themselves. This trait may make individuals who perceive high levels of social support more inclined to exhibit their moral qualities and more sensitive to opportunities to showcase those qualities (Winterich et al., 2013), and thus exhibit more prosocial behavior. Drawing upon the aforementioned theoretical underpinnings and relevant empirical findings, we posit the following hypothesis that an individual’s moral sensitivity serves as a mediating variable in the association between their perceived social support and their prosocial behavior (H3).

### 1.4. The chain mediating role of moral identity and moral sensitivity

Moral identity has been established as a critical factor in enhancing moral sensitivity, as demonstrated by previous research (Xiang et al., 2020). Aquino et al.’s (2009) social cognitive model of moral behavior postulates that the degree of internalization of moral identity is directly proportional to the prominence of moral schemas in the self-concept, leading to greater accessibility of these schemas in cognitive processes. Consequently, individuals with higher internalization of moral identity are more adept at using existing moral schemas to interpret their own and others’ behavior from a moral standpoint, resulting in heightened moral sensitivity. Sparks (2015) has further corroborated this model by showing that internalization of moral identity is positively linked to moral sensitivity. Moreover, empirical evidence suggests that perceived social support significantly correlates with actual acquired social support behaviors (French et al., 2018). The provision of social support behaviors fosters morally relevant social experiences of helping others (Heerde and Hemphill, 2018). The significance of moral schemas to oneself is amplified by the acquisition of experiences that hold moral importance (Aquino and Reed, 2002). This, in turn, reinforces the internalization of moral identity by the individual. Additionally, studies have demonstrated that individuals with high perceived social support exhibit greater confidence (Freeman and Rees, 2010), which leads to an increased desire to present themselves. This trait may result in individuals with high perceived social support being more inclined to showcase their moral qualities and possess higher moral identity representations. Furthermore, individuals with high moral identity representations tend to be more enthusiastic about exhibiting their moral qualities and gaining recognition from others (Jennings et al., 2015). They are also more alert to opportunities to demonstrate their moral qualities (Winterich et al., 2013), and as a result, are more sensitive to identifying moral issues in the given situation. Consequently, individuals with high perceived social support experience a higher degree of internalization of moral identity, which in turn leads to greater sensitivity to moral problems in their surroundings and more participation in prosocial behavior. Hence, based on these findings, this study proposes research hypothesis that suggests moral identity and moral sensitivity play a chain mediating role between perceived social support and prosocial behavior (H4).

**Figure 1:**
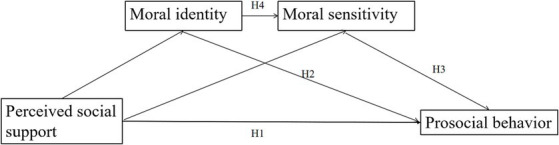
Hypothetical model of the relationship between perceived social support, moral identity, moral sensitivity, and prosocial behavior.

To sum up, the present investigation aims to conduct a thorough analysis of the manner in which college students’ prosocial behavior is influenced by perceived social support, moral identity, and moral sensitivity, taking into account the potential pathways of interaction among these factors. Additionally, we have incorporated age and gender as control variables, so as to control for their potential impact on the study findings. Our main research hypothesis posits that perceived social support will have a significant impact on prosocial behavior, which will be mediated both independently by moral identity and moral sensitivity, as well as through their chain mediating role. A visual representation of this hypothetical model is provided in Figure 1.

## 2. Methods

### 2.1. Participants

A convenience sampling method was used to select one university in the eastern, central and western regions of China for administering the questionnaire, and after collecting the results, the data with apparently incomplete answers and apparently inattentive answer process were firstly excluded (We embedded an attention check into the survey that asked participants to “choose the second option.” If the participant selects incorrectly, the data will not be used.), and 978 valid questionnaires were finally collected. Among them, 496 (50.7%) were female participants and 482 (49.3%) were male participants; 329 (33.6%) were freshmen, 325 (33.2%) were sophomores, 170 (17.4%) were juniors, and 154 (15.8%) were seniors. The age range of participants was 18–23 years, with a mean age of 20.49 years (*SD* = 1.71).

### 2.2. Procedure

The research design used in this study was a questionnaire survey. Before participation, participants received information about the study. They were informed that participation was completely volontary and anonymous. Furthermore, it was emphasized that they could withdraw at any time without providing reasons. Ethical approval was granted by the Academic Ethics Committee of School of Humanities, Tongji University. All participants gave informed consent.

Prior to the students completing the scales, our tautological emphasis was on their answering honestly and confidentially, and their anonymity was ensured. Under the guidance of trained investigators, participants completed the following questionnaires: Perceived Social Support Scale, Prosocial Behavior Tendency Scale, Moral Identity Scale, Dispositional Moral Sensitivity Scale. Finally, this study got a final representative sample of 978.

#### 2.2.1. Perceived social support

This questionnaire was developed by Zimet et al. (1988), translated into Chinese by Chou (2000) and tested among Chinese participants, and found to have good reliability and validity for measuring the perceived social support of Chinese participants. The 12-item questionnaire uses a 7-point scale to measure support from family and friends, as well as participants’ perceptions of support from others. In this questionnaire, “1” means “strongly disagree” and “7” means “strongly agree.” The higher the total score, the higher the level of social support experienced by the participant. The overall consistency coefficient of the questionnaire was 0.95, and the sub-dimension coefficients were 0.86, 0.85, and 0.85. The scale validity indicators were well fitted (*χ*^2^/df = 2.38, GFI = 0.91, IFI = 0.92, CFI = 0.90, TFI = 0.91, RMSEA = 0.06).

#### 2.2.2. Prosocial behavior

The Prosocial Behavior Tendency Scale was used (Carlo and Randall, 2002). The scale has been used with Chinese samples and was found to have good construct validity and satisfactory internal consistency (ranging from a = 0.56 to a = 0.83) (Kou et al., 2007). The scale contains 26 items, such as “I often help others when they are in a bad mood,” and uses a five-point Likert scale, ranging from 1 “very unlike me” to 5 “very like me.” “, the higher the score, the higher the individual’s propensity for prosocial behavior. In this study, the Cronbach’s alpha coefficient for the prosocial behavior tendency scale was 0.90. The scale validity indicators fit well (*χ*^2^/df = 2.47, GFI = 0.92, IFI = 0.91, CFI = 0.93, TFI = 0.95, RMSEA = 0.09).

#### 2.2.3. Moral identity

The Chinese version of the Moral Identity Scale (Wang and Yang, 2013), initially developed by Aquino and Reed (2002), was used to assess moral identity. There are 10 items, such as “Having these traits is an important part of my sense of self” and “Being a person with these traits makes me feel good.” A 5-point scale was used, with 1 being “totally disagree” and 5 being “totally agree,” the higher the score, the higher the individual’s moral identity. This scale has demonstrated adequate reliability and validity in a study of Chinese college students (Wang and Yang, 2013). In this study, the Cronbach’s alpha coefficient for the moral identity scale was 0.86, and the scale validity indicators were well fitted (*χ*^2^/df = 3.12, GFI = 0.91, IFI = 0.92, CFI = 0.90, TFI = 0.93, RMSEA = 0.06).

#### 2.2.4. Moral sensitivity

The Dispositional Moral Sensitivity questionnaire developed by Zheng (2008) was used, which contains 28 items, such as “I would be ashamed of myself for not upholding justice.” The scale is scored on a 6-point scale, with 0 indicating complete opposition and 5 indicating complete agreement, and higher scores on, the scale indicate greater moral sensitivity. In this study, the Cronbach’s alpha coefficient of the psychological resilience scale was 0.87. The scale validity index was well fitted (*χ*^2^/*df* = 2.59, GFI = 0.93, IFI = 0.92, CFI = 0.93, TFI = 0.94, RMSEA = 0.06).

#### 2.2.5. Control variables

Considering that previous studies have shown that gender (Eagly, 2009) and age (Spenser et al., 2020) have significant effects on prosocial behavior, the gender and age of the individual were used as control variables in the current study. In the current study, we coded the individual’s gender as a dummy variable.

### 2.3. Common variance test

To avoid common method bias due to individual self-report and to improve the authenticity of participants’ responses, it was firstly stated in the instruction setting of the test questionnaire that this questionnaire will be filled in anonymously, and the answers to the questionnaire will be strictly confidential and the answers will only be for academic research. The results showed that the eigenvalues of 14 factors were greater than 1 in total, and the variance explained by the largest factor was 25.94%, which was less than 40%, indicating that there was no serious common method bias effect in this study.

## 3. Results

### 3.1. Correlation analysis of perceived social support, moral identity, moral sensitivity, and prosocial behavior

Through the correlation analysis of individual prosocial behavior, perceived social support, moral identity and moral sensitivity, it was found that perceived social support was significantly and positively correlated with moral identity, moral sensitivity and prosocial behavior; moral identity was significantly and positively correlated with moral sensitivity and prosocial behavior; and moral sensitivity was significantly and positively correlated with prosocial behavior (see Table 1 for details).

**Table 1:** Descriptive statistics, correlations, and average variance extracted of the main variables.

Variables	*M*	*SD*	1	2	3	4	5	6
1.gender	0.51	0.5	1					
2.age	20.49	1.71	0.03	1				
3. perceived social support	4.22	0.65	0.03	0.06	1			
4. moral identity	3.28	0.60	0.01	0.06	0.33**	1		
5. moral sensitivity	3.42	0.71	0.05	0.05	0.46^**^	0.20*	1	
6. prosocial behavior	4.18	1.44	0.03	0.03	0.20*	0.38**	0.19*	1

Dummy variables are used for gender: male = 1, female = 0; *p < 0.05, **p < 0.01, ***p < 0.001, the same below.

### 3.2. Analysis of mediating effects

This study used SPSS 22.0 with the SPSS macro program PROCESS (Model 6) developed by Hayes (2013) for data analysis (Hayes, 2013). Mediating effects were tested by estimating 95% confidence intervals for mediating effects with 5,000 sample draws, controlling for individual age and gender.

**Figure 2:**
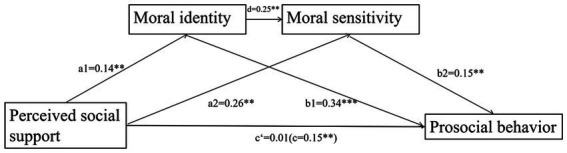
The mediating effects model after controlling for gender and age. **p < 0.01; ***p < 0.001.

The test results showed that perceived social support significantly and positively predicted prosocial behavior (c) and moral identity (a1); both perceived social support (a2) and moral identity (d) positively predicted moral sensitivity. However, when both moral identity and moral sensitivity were included in the regression equation of perceived social support and prosocial behavior, perceived social support could not directly predict prosocial behavior (c’), as detailed in Figure **2**.

The results of the test of mediating effects (see Table 2 for details) indicate that moral identity and moral sensitivity play a significant mediating role between perceived social support and prosocial behavior, with an overall standardized mediating effect value of 0.15. The mediating effect consists specifically of indirect effects resulting from three paths: indirect effect 1 formed by the path of perceived social support→moral identity→prosocial behavior (effect value of 0.10); indirect effect 2 formed by the path of perceived social support→moral identity→prosocial behavior (effect value of 0.03); indirect effect 3 formed by the path of perceived social support→moral sensitivity→prosocial behavior (effect value of 0.02); and indirect effect 3 formed by the path of perceived social support→moral identity→moral sensitivity→prosocial behavior (effect value of 0.02). The indirect effect of perceived social support, moral identity, and moral sensitivity to prosocial behavior was 0.02. The 95% confidence intervals for these indirect effects did not contain 0, indicating that they were all significant.

**Table 2:** Mediating effects of moral identity and moral sensitivity.

Mediation Path	Standardized mediated effect value	95%CI
Path1 (a1 × b1)	0.10	(0.01, 0.03)
Path2 (a2 × b2)	0.03	(0.02, 0.06)
Path3 (a1 × d × b2)	0.02	(0.01, 0.02)
Total mediating effect	0.15	(0.07, 0.11)

Path 1: perceived social support→moral identity→prosocial behavior; Path 2: perceived social support→moral sensitivity→prosocial behavior; Path 3: perceived social support→moral identity→moral sensitivity→prosocial behavior.

## 4. Discussion

This investigation aimed to analyze the impact of perceived social support on prosocial behavior. Additionally, it explored the mediating role that moral identity and moral sensitivity play in the connection between perceived social support and prosocial behavior. The findings of the research indicated that there is a positive and significant correlation between perceived social support and prosocial behavior. It also demonstrated that perceived social support is capable of influencing prosocial behavior not only by an independent mediating function of moral identity and moral sensitivity, but also by a chain mediating role of moral identity and moral sensitivity. The research supports the chain mediation model of perceived social support influencing prosocial behavior and provides evidence to investigate the internal mechanisms through which college students’ perceived social support affects their prosocial behavior.

### 4.1. Perceived social support promotes prosocial behavior among college students

Perceived social support experienced by individuals themselves is more effective in predicting prosocial conduct than objective social support received from the outside world. The findings of this study provide credence to the notion that prosocial conduct is favorably predicted by perceived social support. The importance of self-perceptions in social support is highlighted by subjective social support, which better captures internal emotions and measures how happy people are with the availability of assistance and with external support. People specifically report higher levels of perceived social support when they are satisfied with their relationships and environment and have access to more care, respect, interpersonal resources, and emotional support from a social network made up of parents, friends, and other people (Chou, 2000). The study emphasizes that perceived social support is a crucial component of individual psychological resources, which can help individuals cope with stress (Yildirim and Green, 2023) and negative emotions, and improve resilience (Yildirim and Tanrıverdi, 2021; Yildirim et al., 2023), social adjustment. Meanwhile, perceived social support exerted a statistically significant influence on the emotional well-being (Yildirim and Green, 2023). Overall, the study highlights the importance of perceived social support in facilitating prosocial behavior, and underscores the significance of individual psychological resources in promoting positive external behaviors. The likelihood of individuals exhibiting prosocial behavior is positively correlated with their perceived social support. The buffer effect hypothesis, proposed by Cohen and Wills (1985), suggests that high levels of perceived social support can lead to positive perceptions, effective stress-coping mechanisms, and the development of adaptive social networks in college students. These factors provide a solid foundation for the emergence of prosocial behaviors. It is important to note that the perception and utilization of social support plays a more significant role in this relationship compared to objective social support. This means that the way individuals perceive and use social support can directly influence their likelihood of exhibiting prosocial behavior. Therefore, it can be concluded that perceived social support can significantly encourage college students to display more prosocial behaviors.

### 4.2. The mediating role of moral identity and moral sensitivity

The influence of perceived social support on the prosocial behavior of college students can be explained through the individual mediating roles of moral identity and moral sensitivity.

Previous study (Hertz and Krettenauer, 2016) has shown that perceived social support can affect the mediating role of moral identity, leading to an increase in prosocial behavior tendencies. This finding is consistent with previous research (Hardy et al., 2017), which suggests that individuals with a higher level of moral identity are more likely to exhibit prosocial behavior. This study highlights the importance of moral identity as a personality trait that can influence the prosocial behavior tendencies of college students. Individuals with high moral identity are more likely to maintain a supportive and friendly attitude toward others and strive to have a positive impact on society (Kaur, 2020). Additionally, the bidirectional processing theory of empathy proposed by Goubert et al. (2005) suggests that empathy is generated by a combination of bottom-up emotional sharing processes and top-down cognitive coordination control processes. This theory further supports the notion that moral identity plays a crucial role in shaping the prosocial behavior tendencies of college students. The recognition of moral virtues motivates college students to advocate for the expansion of their moral sphere of concern through top-down approaches. This involves the incorporation of internalized or symbolic moral standards when processing information from others, as suggested by Kaur (2020). Additionally, by perceiving similarities between themselves and the recipient, as proposed by Lee et al. (2014), students develop empathy toward the recipient, ultimately promoting prosocial behavior.

According to the Broaden-and-build theory of positive emotions, individuals who perceive high levels of social support tend to be more confident (Freeman and Rees, 2010). The acquisition of confidence makes individuals more likely to engage in self-promotion. This characteristic may lead individuals who perceive high levels of social support to be more inclined to exhibit their moral qualities and demonstrate higher levels of moral identity. In other words, perceiving high levels of social support can enhance moral identity, individuals are more likely to engage in prosocial behavior. Consequently, perceived social support can strengthen the internalization and representation of individuals’ moral identity, thus demonstrating more prosocial behavior. Therefore, high levels of moral identity can promote positive social attribute behaviors in prosocial behavior situations to positive qualities of individuals, which can enhance the influence of perceived social support on prosocial behavior and motivate individuals to imitate and learn moral behaviors. College students with high level of perceived social support, they will pay more attention to and maintain the positive emotions in their daily lives, and are more willing to imitate the initiators of prosocial behaviors to provide helpful support to others when they experience a sense of moral elevation and moral identity.

The impact of perceived social support on prosocial behavior can be understood through the mediating role of moral sensitivity. Research has established that heightened moral sensitivity aids in the recognition and processing of moral and moral dilemmas, thereby fostering moral behavior. Conversely, reduced or absent moral sensitivity can lead to moral apathy and ignorance. (Fowler et al., 2009; Pedersen, 2009). In accordance with the perspective of social information processing theory, individuals spontaneously interpret new information based on their memory of associated experiences, including semantic concepts and schemas they have developed. The process of perceiving social support entails the recognition of respect, care, and assistance from family, friends, or other groups; this process involves a plethora of concepts and schemas regarding interpersonal interaction and the provision or denial of support (Wyer and Srull, 1986; Mankowski and Wyer, 1997). Conversely, moral problems typically arise in the context of interpersonal relationships (Jia and Krettenauer, 2017) and necessitate the application of concepts and schemas that are related to interpersonal interactions. Consequently, social information processing theory posits that the concepts and schemas derived from perceived social support, which pertain to support, rejection, harm, and help in interpersonal interactions, can furnish individuals with prior experiences that inform their judgment of moral issues. This, in turn, can heighten moral sensitivity and facilitate the performance of prosocial behaviors. Additionally, research has shown that individuals who perceive high levels of social support are more confident (Freeman and Rees, 2010), and the acquisition of confidence makes them more likely to showcase themselves. This trait may make individuals who perceive high levels of social support more inclined to exhibit their moral qualities and more sensitive to opportunities to showcase those qualities (Winterich et al., 2013), and thus exhibit more prosocial behavior.

### 4.3. The chain mediating role of moral identity and moral sensitivity

The impact of perceived social support on an individual’s prosocial behavior can be attributed to its impact on moral identity and moral sensitivity. The outcomes of the study indicate a strong correlation between moral identity and moral sensitivity, which corroborates the findings of earlier research to a certain extent (Aquino et al., 2009). The relationship between moral identity and moral sensitivity can be accounted for in two ways. Firstly, the social cognitive model suggests that a higher degree of internalization of moral identity leads to greater importance being placed on moral schemas in the self-concept. This in turn makes them more accessible in the cognitive process, thereby enabling individuals to use existing moral schemas more frequently and efficiently in explaining their own and others’ behavior from a moral standpoint, resulting in greater moral sensitivity (Aquino et al., 2009). The acquisition of socially supportive behaviors offers a social experience of aiding others, which has moral significance (Heerde and Hemphill, 2018). This moral experience improves the importance of moral schemas to the self, thereby strengthening the individual’s internalization of moral identity (Aquino and Reed, 2002). Secondly, studies suggest that individuals with high perceived social support exhibit greater confidence (Freeman and Rees, 2010). This confidence acquisition leads them to become more willing to showcase themselves. People with high perceived social support are more likely to manifest their moral qualities and have greater moral identity representation. Furthermore, individuals with high moral identity representation are more inclined to display their moral qualities and gain recognition from others, making them more sensitive to opportunities to exhibit their moral qualities (Winterich et al., 2013; Jennings et al., 2015). Therefore, individuals with high perceived social support internalize moral identity to a greater degree, rendering them more sensitive to moral issues in the context and increasing their engagement in prosocial behavior.

### 4.4. Research implications and limitations

This study establishes the chain mediation effect, demonstrating how perceived social support, moral identity, and moral sensitivity interact to influence college students’ prosocial behavior. This finding addresses the limitations of previous research that has only examined the impact of perceived social support on prosocial behavior. The implications of this study are practical, as it provides a means for enhancing prosocial behavior. Specifically, the study suggests that improving perceived social support and developing moral identity and moral sensitivity can enhance individuals’ adaptability and coping skills, promoting prosocial behaviors such as coordination of interpersonal relationships, helpfulness, and protection of public interests. In addition, for educators and mental health professionals, understanding the relationship mechanism between perceived social support and prosocial behavior is also beneficial for them to find the right approach in the education process. Finally, based on the findings of this study, the nature of how perceived social support affects prosocial behavior may lie in individuals’ moral interpersonal relationships and psychological resources. Institutions such as schools and communities can enhance individuals’ moral identity, moral sensitivity, and positive emotions through psychological training. This can be used to expand individuals’ positive psychological resources and strengthen their prosocial behavior.

However, the study’s cross-sectional and correlational design precludes causal inference, indicating a need for future longitudinal or experimental studies. Additionally, the use of self-reported measures by college students may introduce bias, and future studies should use alternative methods, such as other-rated measures, to obtain more objective information and reduce social desirability.

## 5. Conclusion

Perceived social support is significantly and positively related to prosocial behavior among college students, and high perceived social support promotes individuals to exhibit more prosocial behavior. Moral identity and moral sensitivity play a chain mediating role in the relationship between perceived social support and prosocial behavior. Perceived social support can not only influence prosocial behavior through the independent mediating role of moral identity and moral sensitivity, respectively, but also influence prosocial behavior through the chain mediating role of moral identity and moral sensitivity.

## Data availability statement

The raw data supporting the conclusions of this article will be made available by the authors, without undue reservation.

## Ethics statement

The studies involving humans were approved by the Academic Ethics Committee of School of Humanities, Tongji University. The studies were conducted in accordance with the local legislation and institutional requirements. Written informed consent for participation in this study was provided by the participants’ legal guardians/next of kin.

## Author contributions

QL conceived the paper, ran statistical analyses, conducted the experiments, and contributed to the manuscript. GH conceived the paper and contributed to the manuscript. All authors have read and agreed to the published version of the manuscript.
